# Gastrocolic fistula, a rare complication

**DOI:** 10.1259/bjrcr.20170121

**Published:** 2018-05-31

**Authors:** Farah Aslam, Nabil El-Saiety, Abdus Samee

**Affiliations:** 1 Department of General Surgery, Newham University Hospital, Barts Health NHS Trust, London, UK; 2 Department of Radiology, Royal Oldham Hospital, Pennine Acute Hospitals NHS Trust, Manchester, UK

## Abstract

A 77-year-old male presented with diarrhoea, weight loss and faeculent vomiting. CT scan identified a stricturing lesion in the transverse colon. The man, however, had no features suggestive of large bowel obstruction. This unusual presentation of faeculent vomiting raised a suspicion of a possible communication between the colon and stomach. A subsequent CT scan with oral contrast confirmed the presence of a gastrocolic fistula. During explorative laparotomy, a transverse colonic tumour communicating into the stomach via the gastrocolic fistula was detected. The patient underwent an extended right hemicolectomy and distal gastrectomy as a palliative measure. A gastrocolic fistula is a rare, yet important find and should be recognized as a possible sequel of this disease process.

## Clinical presentation

A 77-year-old male presented with a 7 day history of “dark coloured faeculent” vomitus and diarrhoea. He further reported unintentional weight loss of 7 kg over the last 4 months, lethargy and being “unwell”. He denied any abdominal pain or distension. His medical history included chronic hyponatraemia, myocardial infarction, Type II diabetes mellitus and sigmoid diverticular disease.

His observations on admission were a temperature of 36.2°, respiratory rate of 22 with saturation of 97% on air, heart rate of 63 beats per minute and a blood pressure of 109/51 mmHg. Abdominal examination revealed a soft, non-distended, non-tender abdomen with mild fullness over the caecal pole. There were no signs of peritonism. Per rectal examination revealed an empty rectum with no evidence of a mass or bleeding. The faeculent vomiting prompted a surgical opinion leading to a series of investigations.

## Differential diagnosis

Based on the history and presenting symptoms, our differential diagnosis included acute or subacute intestinal obstruction secondary to a mechanical, inflammatory or neoplastic pathology.

## Investigations/Imaging findings

Blood tests revealed a microcytic anaemia with haemoglobin of 76 g l^−1^ and mean corpuscular volume of 84 fl. His white blood cell count was 11.4 × 10^9^ l^−1^, CRP 5.2 and platelet of 372 × 10^9^ l^−^^1^. Urine and electrolytes revealed low sodium of 128 mmol l^−1^ in keeping with chronic hyponatraemia. A low serum albumin of 29 g l^−1^ was found, as expected in locally advanced colon cancer. The coagulation profile, lactate level and CEA were normal. Both urine dipstick and stool sample were also negative.

**Figure 1.**
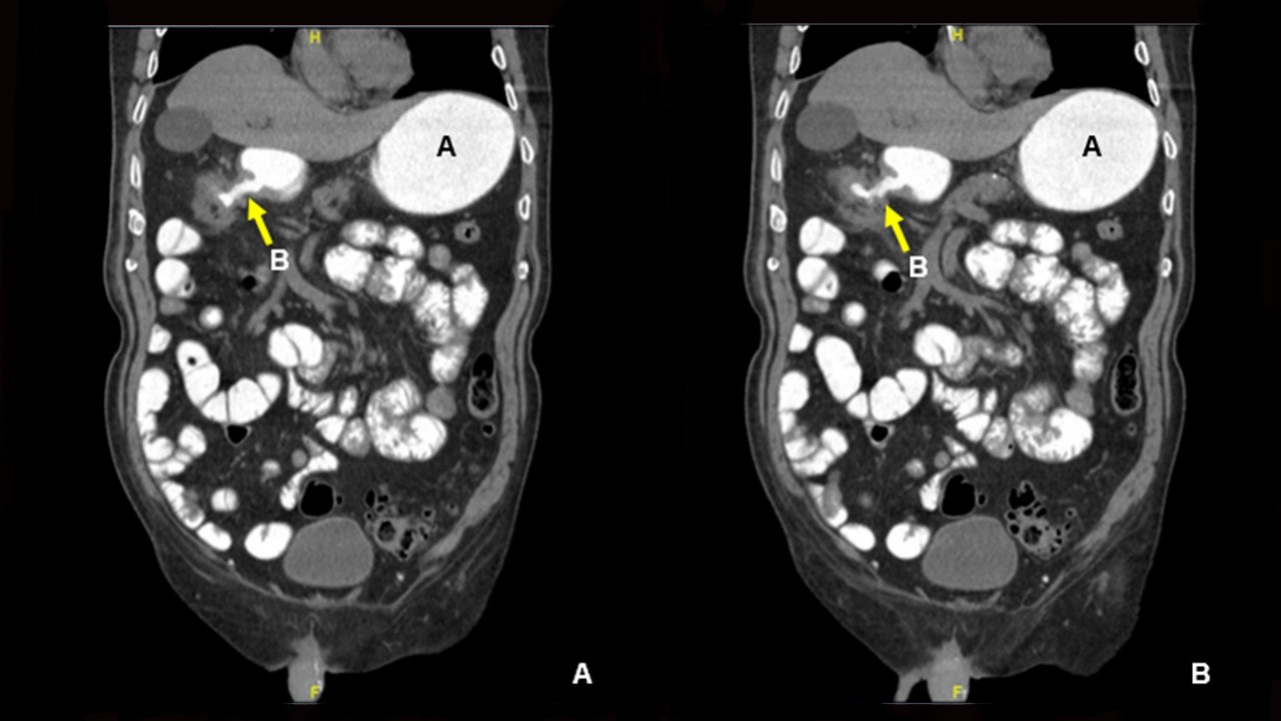
Coronal contrast CT scan images of abdomen demonstrates a dilated stomach (A) and gastrocolic fistula (B).

**Figure 2.**
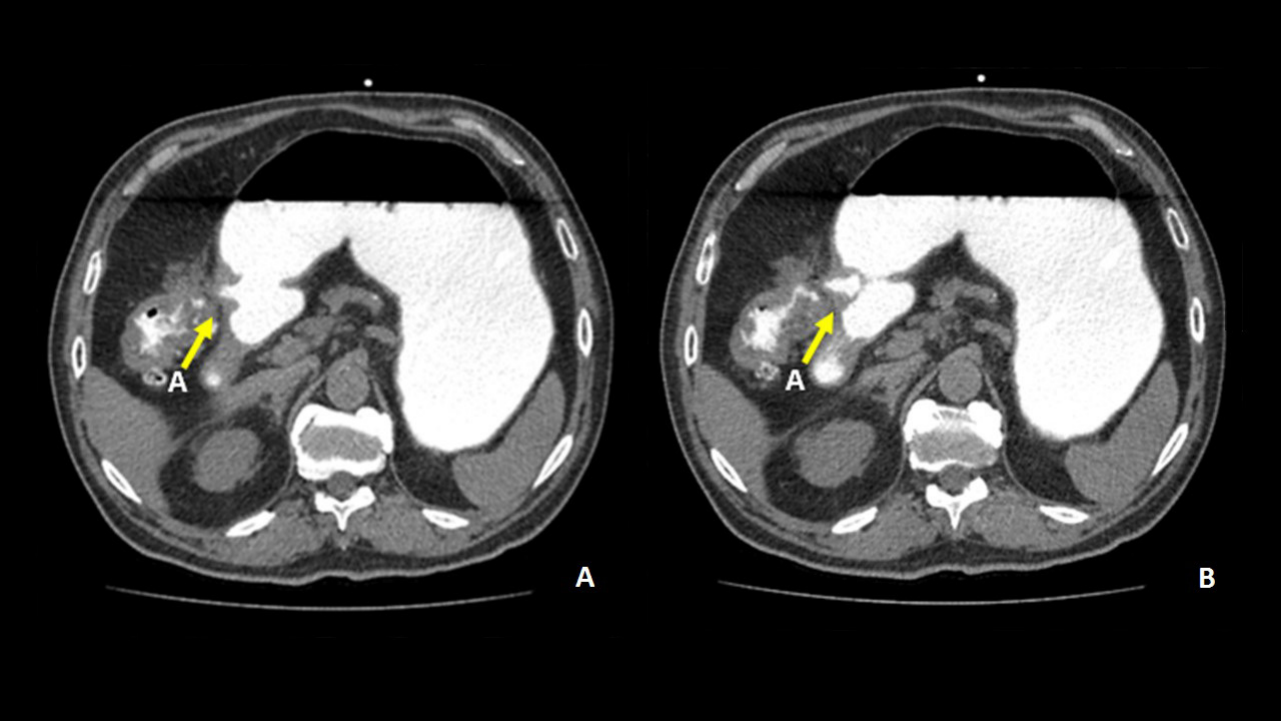
Axial CT scan images demonstrating oral contrast communicating between stomach pylorus and proximal transverse colon (A).

An oesophagogastroduodenoscopy revealed duodenitis with no evidence of acute or recent stigmata of bleeding. The initial CT scan with intravenous contrast revealed a stricturing mass in the proximal transverse colon in close proximity to the pylorus of the stomach. Further examination with oral contrast demonstrated a distended stomach and a communication between the pylorus and transverse colon, thus confirming the presence of a gastrocolic fistula (Figures 1a,b and 2a,b). Imaging did not reveal small bowel dilatation.

## Treatment

The patient was subsequently transferred to a regional specialist centre for further optimization and investigations including a staging CT scan that reported T4 N0 MX disease with no radiological evidence of distant spread. Following multidisciplinary team meeting discussion (MDT), he underwent a distal gastrectomy, extended right hemi-colectomy with Roux-en-Y reconstruction. Intraoperatively, there was no evidence of metastatic disease.

Histology confirmed a Duke’s B (T4b, N0, MX) moderately differentiated adenocarcinoma of the transverse colon with no evidence of extramural vascular invasion.

10 days post-laparotomy, the patient developed a duodenal stump leak and therefore, underwent exploration, repair of the leak, and a feeding jejunosotomy.

## Follow up and Outcome

The patient had a prolonged stay in the hospital needing organ and nutritional support. He has now made a full recovery.

## Discussion

A gastrocolic fistula is an abnormal communication between a segment of large bowel (usually distal) with a portion of the stomach, most commonly the greater curvature.^1^ It is a rare find with only a handful of cases being reported in existing literature. A greater incidence has been reported in females between the ages 50–60 years.^2^

First described in 1755 by Albrecht von Haller, a gastrocolic fistula may result secondary to various pathologies including chronic pancreatitis, diverticular disease, malignancies of the stomach or colon or tumours invading the biliary tract, pancreas and duodenum.^2, [Bibr b2]^ ^3^ The patient may present with an array of signs including anaemia, weight loss, malaena, ascites, palpable mass, faeculent vomiting or faecal oris.^4, 5^ There is, however, a well-known triad of diarrhoea, weight loss and faeculent vomiting which in fact our patient displayed.^6^ Whether the cause of a gastrocolic fistula is benign or malignant, the process of formation of fistula is thought to be due to a prolonged inflammatory response resulting in migration of intestinal epithelial cells into deeper layers of the intestinal wall causing localized tissue damage and subsequently a communication to other organ or the body surface.^5, 7^

The potential risk factors for this clinical presentation include helicobacter pylori status, tumour genetics and microsatellite instability and should be further investigated if necessary.^8^ Examination with gastroscopy may fail to identify the abnormal fistulous tract opening. Colonoscopy may fail to assess the pathology, progress beyond the stricture itself or identify the tract. In addition, biopsies, although key to establishing the diagnosis, may be inconclusive due to technical reasons.^2^

The presence of frank faeculent vomiting in a soft and non-distended abdomen raised a strong possibility of gastrocolic fistula in our patient. This was eventually confirmed via oral contrast studies. Other imaging methods to demonstrate gastrocolic fistula include a barium enema study.^9^ Further management is discussed at MDT. In patients deemed suitable for resection, an en bloc resection of the tumour remains the mainstay of treatment. Additional neoadjuvant treatment is tailored per the histology and the individual’s needs. The main prognostic factors include the age of the patient, the stage of disease at time of diagnosis and the tumour genetics. These patients unfortunately have a poor prognosis with the mean survival of less than 2 years.^10^

Our case demonstrate a rare but important complication of a common large bowel pathology. Faeculent vomiting is usually observed in intestinal obstruction resulting in a distended abdomen. Our patient, however, had a completely soft, non-distended and non-tender abdomen. In addition, he did not have any risk factors for this pathology. The faeculent vomiting, in the absence of an obstructed colon raised a strong suspicion of gastrocolic fistula subsequently confirmed via imaging studies. These clinical findings, hence, are important for clinicians to recognize enabling early diagnosis and appropriate treatment.

## Learning points

1. Patients presenting with faeculent vomiting in absence of signs of obstruction raise suspicion of a gastrocolic fistula.
2. Radiological studies such as CT scan of the abdomen with intravenous and oral contrast is an extremely valuable tool for confirming the diagnosis.
3. Management is tailored individually and based upon discussion via a MDT.
